# Cementing the bricks

**DOI:** 10.1172/JCI173350

**Published:** 2023-07-17

**Authors:** Sneha P. Rath

**Affiliations:** Department of Molecular Biology, Massachusetts General Hospital; Department of Systems Biology, Harvard Medical School; and Broad Institute of MIT and Harvard, Cambridge, Massachusetts, USA.

This is a story of a second-year graduate student who gets excited about a paper related to her slowly-but-surely emerging PhD thesis. The paper had reported that knocking out a certain human gene suddenly triggered innate immune response — an alarm that was previously thought to be reserved only for viral, i.e., “foreign,” invasion. She recognizes that the paper’s finding is transformative, because it expands the scope of what a cell’s defense system can sense besides infections. She feels curious and inclined to follow up on the many questions that storm her mind: What is the purpose and mechanism of such an immune response?, Could it be targeted to treat autoimmune disorders?, and so on. With the excitement of possibilities and the tenacity of a dedicated student, she embarks on reproducing the result so that she can continue to build on it. But before the next brick is laid, the wobbly brick that she had stepped on topples her. The result fails to reproduce. Hypotheses are lost to a cloud of confusion.

Doubt and despair settle in. A door is slammed shut.

A young scientist who is excited to build on an existing discovery neither wishes nor anticipates that the prior discovery itself is false. In the dull silence that lingers an unexpected ethical dilemma weighs heavily on her: should she forget about the unexplainable, irreproducible nugget of literature that she discovered and move on to something else, or should she invest the indefinite time it may take to clarify and correct this literature?

Her choice is not easy. On one hand, it is advisable for a fledgling scientist like her to work on phenomena that are robust and real. On the other hand, irreproducible results can offer the most important and memorable lessons. They expose layers of systematic issues in the scientific enterprise: unexpected gaps in knowledge, dangerously perpetuating mistakes, delicate vulnerabilities of the chosen lens of investigation, and imperfections of peer review. She realizes that making new discoveries — laying down new bricks — is a joy of being a scientist. But righting the wrong — fixing the wobbly bricks that we stumble upon — is a responsibility that all scientists share, including those in training.

She feels emboldened and excited to get to the bottom of the matter. She returns to the published paper and peruses it with great care. “The devil must be in the details.” Sure enough, she finds that the reagent used to knockout the gene had a certain modification that was placed as a routine practice for increasing bioavailability. However, the generic control reagent was missing this supposedly neutral modification. She realized that this was a classic case of an overlooked, missing, and mismatching control. So she forged a new hypothesis: it must be the presence or absence of this modification, not the reported gene, that modulates immune response. She set out to test a full suite of carefully designed controls in multiple, independent experiments and, to her shock and relief, confirmed her hypothesis. After the two grueling but steadfast years since the initial fallout, she had not only corrected the previous claim but also published about the real culprit that can set off our cell’s immune system.

What does this student’s path teach us, and how can we support more journeys like hers? At its core, this story reminds us that while seemingly “great” discoveries are captivating, dogma changing, eye opening, thought provoking, and inspiring, the truly greatest discoveries are immutable. This badge of immutability is not passively earned but can be secured only if we encourage and reward critical (re)investigation.

As a member of the scientific community myself, I would like to leave you with two parting thoughts for a future that I hope we will build together.

(a) As scientists, let us equip future explorers with all the information they would need to faithfully reproduce our work decades, or even centuries, later. To reward reproducibility, we could use a metric akin to number of citations, except this would be a score for a figure/paper based on how many others are able to reproduce it. Such an endorsement system could also have a monitored forum for open dialogue among active researchers — this would keep publications “alive” and expand the scope of peer review, heightening accountability. In an ecosystem where a publication is not blindly revered as an ultimatum, no graduate student will ever feel isolated or burdened if they are unable to reproduce a result and would have a platform to consult and contribute.

(b) As mentors, let us encourage our trainees to make minimal assumptions about truth and always pause to verify the stability of key bricks at the foundation. Let us view the published body of science not as static facts but pieces of a grand puzzle that we are constantly, collectively, and critically reorganizing.

## Version 1 07/17/2023

Electronic publication

